# Inflammation, neurodegeneration and protein aggregation in the retina as ocular biomarkers for Alzheimer’s disease in the 3xTg-AD mouse model

**DOI:** 10.1038/s41419-018-0740-5

**Published:** 2018-06-07

**Authors:** Alfonso Grimaldi, Carlo Brighi, Giovanna Peruzzi, Davide Ragozzino, Valentina Bonanni, Cristina Limatola, Giancarlo Ruocco, Silvia Di Angelantonio

**Affiliations:** 1 Center for Life Nanoscience, Istituto Italiano di Tecnologia, Rome, Italy; 2 Department of Physiology and Pharmacology, Sapienza University, Rome, Italy; 3 IRCCS Neuromed, Pozzilli, Italy; 4 Department of Molecular Medicine, Sapienza University, Rome, Italy; 5 Department of Physics, Sapienza University, Rome, Italy

## Abstract

Alzheimer's disease (AD) is the most common cause of dementia in the elderly. In the pathogenesis of AD a pivotal role is played by two neurotoxic proteins that aggregate and accumulate in the central nervous system: amyloid beta and hyper-phosphorylated tau. Accumulation of extracellular amyloid beta plaques and intracellular hyper-phosphorylated tau tangles, and consequent neuronal loss begins 10–15 years before any cognitive impairment. In addition to cognitive and behavioral deficits, sensorial abnormalities have been described in AD patients and in some AD transgenic mouse models. Retina can be considered a simple model of the brain, as some pathological changes and therapeutic strategies from the brain may be observed or applicable to the retina. Here we propose new retinal biomarkers that could anticipate the AD diagnosis and help the beginning and the follow-up of possible future treatments. We analyzed retinal tissue of triple-transgenic AD mouse model (3xTg-AD) for the presence of pathological hallmarks during disease progression. We found the presence of amyloid beta plaques, tau tangles, neurodegeneration, and astrogliosis in the retinal ganglion cell layer of 3xTg-AD mice, already at pre-symptomatic stage. Moreover, retinal microglia in pre-symptomatic mice showed a ramified, anti-inflammatory phenotype which, during disease progression, switches to a pro-inflammatory, less ramified one, becoming neurotoxic. We hypothesize retina as a window through which monitor AD-related neurodegeneration process.

## Introduction

Alzheimer’s disease (AD) is the most common form of dementia, accounting for 60–70% of all dementia cases. AD is a progressive neurodegenerative disorder causing irreversible deterioration in cognitive functions, secondary to neuronal cell death and brain atrophy^1,2^. The main pathological and diagnostic features are the accumulation of two proteins: amyloid-β (Aβ) peptide, generated from the amyloid precursor protein (APP), which aggregates into extracellular plaques, and hyper-phosphorylated tau (pTau), which forms intracellular neurofibrillary tangles. Evidence now implies that neuropathological changes involving the accumulation of Aβ occur up to 10–15 years prior to the emergence of clinical symptoms, emphasizing the importance of developing new methods for early diagnosis^3^. Currently, diagnosis is based primarily upon cognitive assessments of patients presenting symptomatic features of cognitive and behavioral changes^4,5^. Computed tomography (CT) and/or magnetic resonance imaging (MRI) are frequently used in the initial diagnosis of cases where cognitive AD signs are present, whereas positron emission tomographic (PET) amyloid imaging is less frequently included, mainly where confirmation is needed^6^. However, PET is not applicable for use in population-wide screening, owing to the relatively high cost of this test. Besides PET amyloid imaging, cerebrospinal fluid (CSF) pathophysiological markers, including Aβ1–42, total tau, and pTau, have also shown high specificity in confirming AD pathophysiology. CSF biomarkers in AD and mild cognitive impairment have been increasingly used showing that lower CSF levels of Aβ42 and higher CSF levels of total tau and pTau compared with controls were associated to severe AD^7^. However, lumbar puncture is an invasive analysis, and its use varies between countries. A desirable matrix for AD biomarker detection could be through plasma, however no significant differences of the concentration of Aβ markers in AD and control subjects have been found between plasma or serum^7^.

Moreover, on the therapeutic side, clinical trials of AD drugs have been hindered, in part, by the limited number of biomarkers that can accurately diagnose this disease, and reliably monitor disease progression and response to treatment. In this framework currently available biomarkers are expensive, invasive, or both, and have been concentrated on brain and CSF targets.

The retina and the brain have been demonstrated to be associated to over a range of neurological diseases and respond similarly to neuropathological conditions^8^. Despite its peripheral location, the retina is part of the central nervous system. Retinal cells are arranged in different layers: the outer layer (OL) containing photoreceptors and the inner layer (IL) containing various cell types: interneurons and retinal ganglion cells (RGC), which send their axons to the thalamus. Glial cells are also found in the retina; astrocytes and microglia are usually restricted to the RGC layer and participate in the control of tissue homeostasis (Supplementary Fig 1). Visual deficits and pathological changes have been described in the retinas of AD patients; indeed, retinal abnormalities such as extensive loss of retinal ganglion cell neurons (RGC)^9,10^, reduced thickness of retinal nerve fiber layer (NFL)^11^, and reduced retinal blood flow^12^ have been observed in AD patients.

The advantages of using retinal screening for diagnosing of probable AD rely on low costs, easy visual accessibility of the tissue, and the non-invasive nature of the tests. It has been recently reported that Aβ accumulates in eyes from both human AD and APP/PS1-transgenic mouse models at late disease stage, and retinal plaques have been correlated with plaque load in the brain and vascular abnormalities^13^. However, these changes have been found also in retinal glaucoma^14,15^ and a multifactorial analysis of retinal tissue is needed to use the eye as potential target for AD diagnosis, especially at pre-symptomatic stage. Recent findings also demonstrated the alteration in the level of endogenous retinal Tau of 3 and 6 months old 3xTg mice, and retina neurodegeneration, measured as a reduction in the number of RGC, starting from 6 months of age^16^. However, while all these reports describe AD signs in the retinal tissue of symptomatic mice, no data report up to now, retinal abnormalities in pre-symptomatic mice.

Another important factor in neurodegenerative diseases is the role of neuroinflammation; although neuroinflammatory responses are commonly described in the brain of AD patients and animal models, only few reports describe retinal glia alterations in AD. Retinal glial cells, including astrocytes and microglia, are responsible for the maintenance of the retinal microenvironment, trophic and structural support, regulation of homeostatic functions and an appropriate immune response, fundamental roles for the proper functioning of the retina. Retinal astrocytes, with flattened cell bodies and fibrous radiating processes are not uniformly distributed along the retina, with higher density in the optic nerve region. Indeed, they envelop the optic nerve axons and blood vessels to maintain retinal brain barrier integrity. Increase of glial fibrillary acidic protein (GFAP) immunoreactivity, indicative of astrogliosis, has been reported in the RGC layer of AD patient biopsies and in the late-symptomatic stages of 3xTg-AD mice^17^.

Like in the nervous system, microglia cells are the resident monocytes of the retina, and they are mainly located in plexiform layers and can undergo different activation states and phenotypes, depending on the stimulus they enter in contact with. Resting microglia is continuously surveying parenchyma and, in response to stress conditions, adopts a reactive phenotype, characterized by changes in the expression of pro and/or anti-inflammatory markers, phagocytic activity, and morphology^18^. In the brain of AD animal models, microglia initially reacts against neuronal damage trying to resolve inflammation, displaying an anti-inflammatory phenotype; then during disease progression, microglia acquires and remains in a pro-inflammatory phenotype characterized, among other, by the production of reactive oxygen and nitrogen species, that cause themselves neuronal death. Increased microglia reactivity in the retina has been reported in bioptic tissues of AD patients^19^ and in late-stage animal models^20^.

Thus, the idea of using the eye as an extension of the brain for non-invasive visualization of AD pathology is rapidly growing; however, there is still controversy on the possible biomarkers useful to early diagnose and potentially monitor development and treatment of AD. Here we investigated retinal biomarkers analyzing retinal tissue of 3xTg-AD mouse model in a longitudinal study starting from young, pre-symptomatic mice. We report that in the RCG layer Aβ plaques and pTau tangles, together with cleaved caspase-3 and glia (astrocytes and microglia) activation are detectable in the retina of pre-symptomatic mice, becoming possible biomarkers for AD diagnosis and follow-up.

## Materials and methods

### Animals

Procedures using laboratory animals were in accordance with the Italian and European guidelines and were approved by the Italian Ministry of Health (no. 457/2017–PR) in accordance with the guidelines on the ethical use of animals from the European Communities Council Directive of 20 September 2010 (2010/63/UE). All efforts were made to minimize suffering and number of animals used. The 3xTg-AD mice bearing the human mutations in the genes encoding presenilin 1 (PS1M146V), amyloid precursor protein (APPSwe), and tau (MAPTP301L)^21^, and age-matched wild-type controls 129sv/C57Bl6 (non-Tg) were purchased from Jackson Laboratories (Bar Harbor, ME) and maintained in our animal facility. Experiments were performed at different PNWs (5–10–20 PNWs for pre-symptomatic; 30-32-40 for early-symptomatic; 50–72 for late-symptomatic mice), corresponding to pre, early, and late-symptomatic mice in line with the time course previously reported^22,23^. Results of each group were compared with the corresponding age-matched non-Tg mice.

### Immunofluorescence analysis

Mice at different ages were killed by a chloral hydrate overdose (400 mg/kg, i.p.) and then intracardially perfused with PBS and 4% PFA; brains and eyes were removed and kept in 4% PFA solution; after 16 h, tissues were passed into a 30% sucrose solution and, after precipitation, they were frozen in isopentane at −80 °C. Sections (20–40 μm) were obtained by a Leica cryostat and subsequently treated for immunofluorescence experiments. Briefly, slices were treated 40 min with a warm solution of antigen retrieval (10 mM Na-citrate, 0.05% Tween 20, pH 6.0, 90 °C) to facilitate and increase the exposure of the antigen (when required) and they were then incubated 45 min in a blocking solution (3% goat serum and 0.3% Triton X-100 in PBS). Primary antibodies were then incubated for 16 h at 4 °C in a solution with 1% of goat serum and 0.1% of Triton X-100 at different concentrations (anti-Iba1, Wako #019-19741, 1: 500; anti-cleaved caspase-3, Cell Signaling Technology, Asp175, 1:500; anti-Aβ, Cell Signaling, D54D2, 1:100; anti-GFAP, Novus Biologicals, #NB300-141, 1:500; anti-PHF-Tau, Thermo Scientific, #MN1020, 1:100; anti-Tuj1, Covance, #MMS-435P, 1:500). The day after slices were left 30 min at room temperature and, after three washes in PBS, sections were stained with the fluorophore-conjugated antibody and Hoechst for nuclei visualization for 45 min, mounted and analyzed by means of a confocal microscope (FV10i, Olympus).

### Microglia density analysis

For the analysis of microglia cells density, in slices immunolabeled for Iba1, a well-known microglia marker, images were acquired using an inverted confocal laser scanning microscope (FV10i Olympus) with a ×60 water immersion objective and a z-step of 0.5 µm. Image processing was performed using ImageJ software in order to create maximum intensity projections of z-series stacks. The number of Iba1+ microglia was counted and calculated as cells per volume (mm^3^). Briefly, the number of cells within each acquired field was divided by the area of the slice multiplied by its thickness. The value obtained was multiplied by 10^9^ to get the number of microglial cells present in a mm^3^ of the slice.

### Astrogliosis analysis

Confocal images of GFAP staining were acquired to evaluate the status of astrogliosis. Images were analyzed by Metamorph image analysis software to obtain a z-projection based on the maximal intensity signal. Astrogliosis was quantified as fluorescence intensity: the threshold was adjusted to accurately represent the number of GFAP-positive cell processes and data were expressed as area occupied by fluorescent cells vs total slice area.

### Analysis of neurodegeneration

To evaluate neurodegeneration, we counted the number of cleaved caspase-3-positive cells. For the retina, the whole slice was considered, while for the brain only the hippocampal region was examined. The images acquired with a fluorescence microscope (Nikon Eclipse Ti, ×40 objective) were analyzed with the Metamorph software. The number of cleaved caspase-3-positive cells was then divided by the total number of ganglion neurons within each retina slice (or by the area of the hippocampal region in the case of the brain) and expressed as a percentage.

### Microglia morphological analysis

For morphological analysis of microglia cells, images were acquired using an inverted confocal laser scanning microscope (FV10i Olympus) with a ×60 water immersion objective and a z-step of 0.5 µm with slices immunolabeled for Iba1. Maximal intensity projections of Iba1 confocal images were analyzed to obtain morphological indicators of cell complexity. Image processing was performed using ImageJ software. Only cells whose cell body and processes were fully contained in the slice were included in the analysis. The soma area was determined by drawing a line around the cell body, by using the freehand selection tool. The extent of microglia ramification was quantified by measuring the area circumscribed by the distal ends of each process, using the polygon selection tool. For skeleton analysis, the maximum intensity projection was enhanced to visualize all microglia processes; this was followed by noise de-speckling to eliminate single-pixel background fluorescence. The resulting image was converted to a binary image and then skeletonized, using the corresponding ImageJ plug-in. Total processes, end point, and junction number were calculated from the skeletonized image.

### Microglia cells sorting from mice retinas

Single cell suspension was stained with CD11b PE and CD45.2 APC-eFluor780 anti-mouse antibodies (eBiosciences) and isolated using a FACSAriaIII (BD Biosciences) equipped with a 561 nm and a 633 nm laser and FACSDiva software (BD Biosciences version 6.1.3). Data were analyzed using a FlowJo software (Tree Star). According to the gate strategy showed in Fig. 3d, cells first gated based on forward and side scatter area (FSC-A and SSC-A) plot were then detected for doublet exclusion both in FSC and SSC parameters (height vs. area). CD45.2 APC-eFluor780-positive cells were then sorted based on CD11b PE expression levels. To reduce stress to retina samples, cells were isolated in gentle conditions using a ceramic nozzle of size 100 µm, a low sheath pressure of 19.84 pound force per square inch (psi) that maintain the sample pressure at 18.96 psi and an acquisition rate of maximum 3000 events/s.

### Real-time PCR analysis

Upon sorting, enriched samples were used for total RNA isolation. Briefly, cells were lysed in Trizol (Invitrogen, CA, USA), retrotranscripted, and analyzed by real-time PCR as described in ref. ^24^. Primers used are listed in Suppl Table 1.

## Results

### Retinal and hippocampal neuron degeneration starts at pre-symptomatic stage in 3xTg-AD mice

In AD brain, the onset of neurodegenerative process and neuronal apoptosis has been linked to the caspase-3-mediated cleavage of AD-linked proteins (APP and presenilins), and AD patients exhibited significant increase in synaptic procaspase-3 and active caspase-3 expression levels compared with age-matched controls^25^. We first asked whether signs of apoptotic neurons could be found in the retina and in the brain of 3xTg-AD mice at different stages of the disease. For this purpose, slices of 3xTg-AD mice were analyzed along disease progression, namely at pre-symptomatic (5–20 post-natal weeks, PNWs), early-symptomatic (30–40 PNWs), and late-symptomatic (50–80 PNWs) stages and compared to those from age-matched non-Tg controls. These time windows have been selected because they correspond, respectively, to the appearance of anatomical, electrophysiological, and cognitive signs typical of AD in the brain of this mouse model^22,23^.

**Fig. 1:**
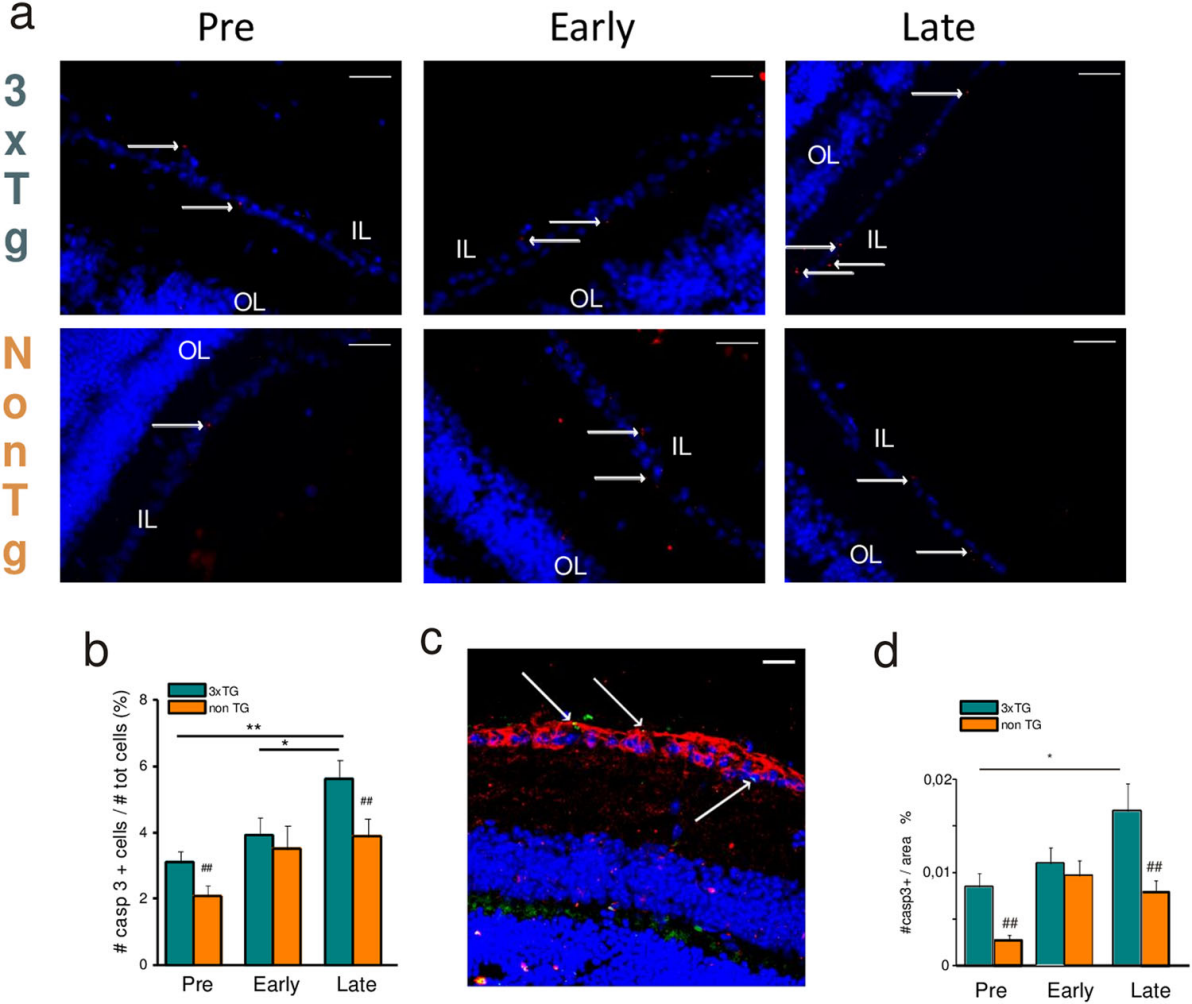
Retinal neurodegeneration starts at pre-symptomatic stage in 3xTg-AD mice. **a** Retinal slices were immunolabeled with anti-cleaved caspase-3 antibody (red) and Hoechst for nuclei visualization (blue) at different ages of 3xTg-AD and non-Tg mice. **b** Percentage of cleaved caspase-3-positive cells (**p < 0.01 pre vs late; *p < 0.05 pre vs early; *p* = 0.12 early vs late; *n* = 16 fields/four slices for each condition; two-way ANOVA, Holm-Sidak) and comparison with age-matched non-Tg mice (^##^*p* < 0.01; *n* = 16 fields/four slices for each condition; two-way ANOVA, Holm-Sidak). **c** Representative image of retinal slice immunolabeled with anti-cleaved-caspase-3 antibody (green), anti Tuj-1 (red), and Hoechst for nuclei visualization (blue). **d** Percentage of cleaved caspase-3-positive cells (**p < 0.001 pre vs late; *p < 0.05 pre vs early; *p* = 0.15 early vs late; *n* = 16 fields/four slices for each condition; two-way ANOVA, Holm-Sidak) and comparison with age-matched non-Tg mice in hippocampal area (^##^*p* < 0.01; *n* = 16 fields/four slices for each condition; two-way ANOVA, Holm-Sidak)

We found, by confocal immunofluorescence analysis, that cleaved caspase-3 punctate staining was present in the RGC layer (Inner Layer; IL) already at five PNWs (Fig. 1a, left red dots). Labeling with an antibody against tubulin isoform βIII (TUJ1), an RGC-specific marker that strongly labels the soma of these neurons, confirmed caspase-3 localization near the cell nucleus (Fig. 1c, green dots). As reported in Fig. 1b, the percentage of caspase-3-positive cells increased during AD progression and was significantly higher respect to what was observed in the retina of age-matched non-Tg mice (Fig. 1a, c).

Despite neuronal loss has been reported in the brain of different AD mouse models starting from 30 PNWs^26,27^, no data are available for the 3xTg-AD model. To compare the results obtained in the retina with brain neuronal loss, brain slices from the same group of mice were analyzed for cleaved caspase-3 expression in the hippocampus. Confocal immunofluorescence analysis revealed that neurodegeneration, measured as cleaved caspase-3 punctate staining, was present in the hippocampus already at five PNWs and displayed similar time course to the one observed in the retina (Fig. 1d).

These results indicated that neuronal apoptosis starts in the AD retina, predominantly in RGC layer, at pre-symptomatic stage and that the time course of neurodegeneration runs parallel to what is observed in the brain.

### Glial activation precedes symptoms onset in brain and retina of 3xTg-AD mice

**Fig. 2:**
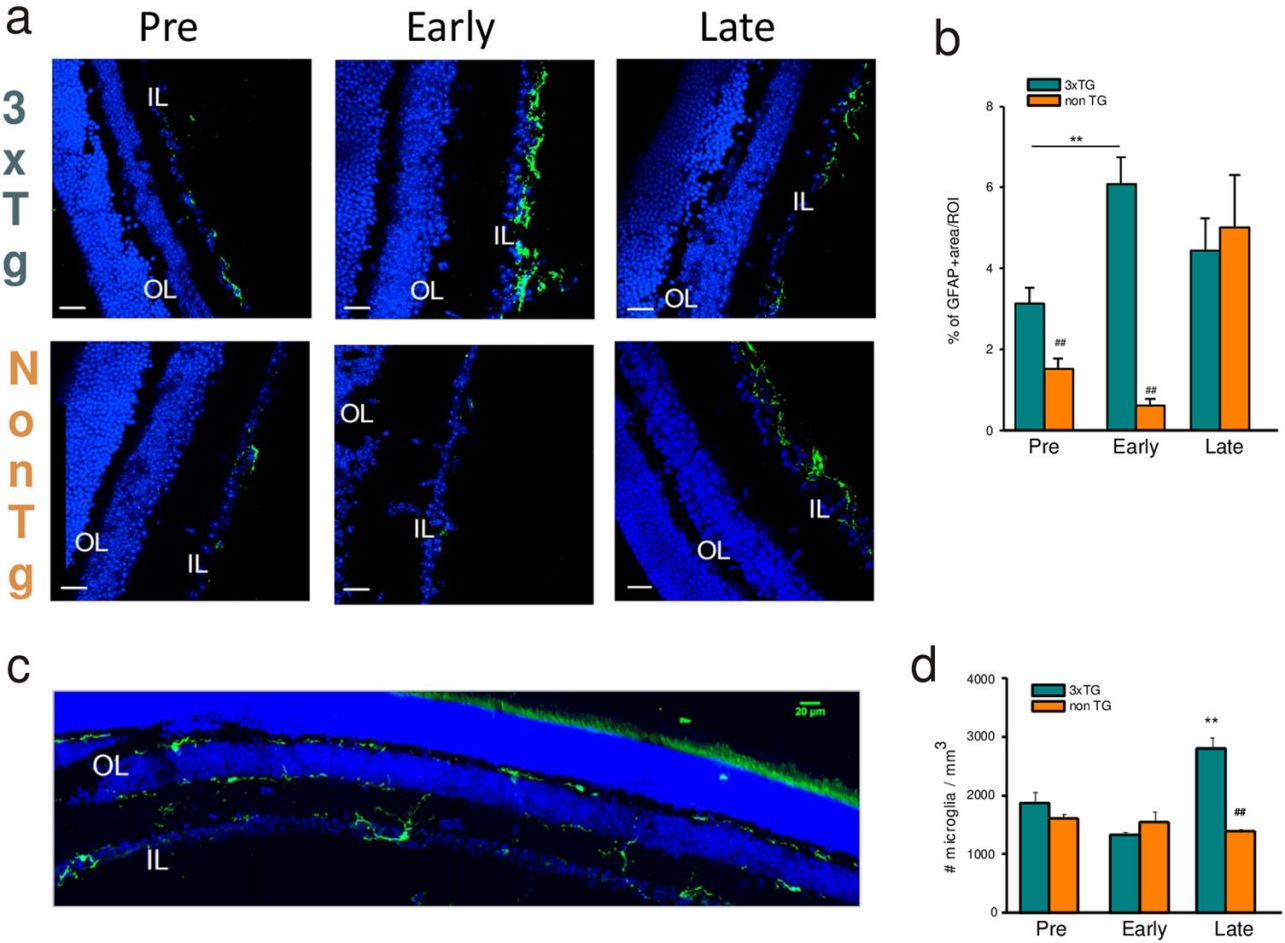
Glial cells density is differently modulated during AD progression. **a** Retinal slices were immunolabeled with anti-GFAP antibody (green) and Hoechst for nuclei visualization (blue) at different ages of 3xTg-AD and non-Tg mice and density of GFAP signal was quantified as shown in **b** (**p < 0.01 pre vs early; *n* = 16 fields/four slices for each condition; two-way ANOVA, Holm-Sidak; ^##^*p* < 0.01 for comparison with age-matched non-Tg mice, two-way ANOVA, Holm-Sidak). **c** Representative multiarea image of retinal slice immunolabeled with anti-Iba1 antibody (green) and Hoechst for nuclei visualization (blue); density of Iba1+ cells was quantified as shown in **d** (**p < 0.01 vs 3xTg-AD pre; ^##^*p* < 0.01 vs age-matched non-Tg mice, *n* = 16 fields/four slices for each condition, two-way ANOVA, Holm-Sidak method for multiple comparison)

Confocal analysis of GFAP staining in the 3xTg-AD mice retina showed marked astrogliosis (measured as fluorescence intensity) already at 5–10 PNWs (Fig. 2a) with reactive astrocytes present in the granular cell layer, as expected. GFAP staining was higher in the 3xTg-AD retina at pre and early-symptomatic AD stages, becoming similar to non-Tg retina at late stage (Fig. 2b).

Immunofluorescence to detect microglia cells (Iba1 positive) in the retina revealed that microglia were mainly found in two layers: the inner plexiform and the outer plexiform layers (Fig. 2c). We found that microglia cell density was similar in the retina of control and 3xTg-AD mice at 5–40 PNWs, while its density strongly increased in late-symptomatic AD mice (Fig. 2d) respect to age-matched controls.

Thus, we deeply investigated microglia phenotype in the retina of 3xTg-AD mice characterizing microglia morphology and activation state-associated transcripts by real-time PCR. Parallel experiments were performed in non-Tg age-matched controls.

**Fig. 3:**
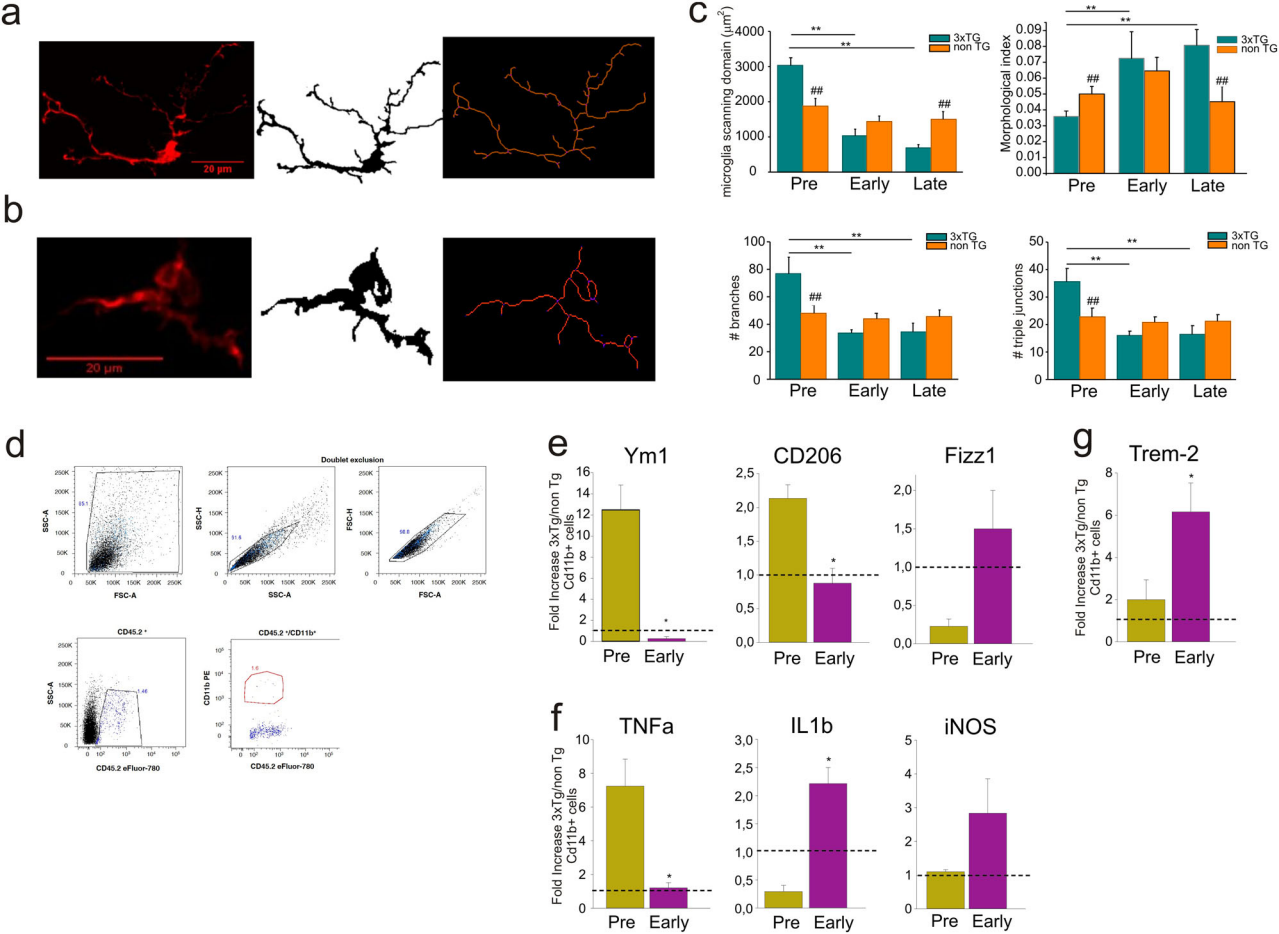
Microglia activation state changes already at pre-symptomatic stage in 3xTg-AD mice. Representative skeletonized images of Iba1+ microglial cell in retinal slices of **a** 3xTg-AD and **b** non-Tg mice. **c** Morphological parameters analyzed for microglia cells at different stages of AD show a more ramified retinal microglia morphology already at pre-symptomatic stage (**p < 0.01 vs 3xTg-AD pre; ^##^*p* < 0.01 vs age-matched non-Tg mice; *n* = 40, two-way ANOVA). **d** Gate strategy for retinal microglia cells sorting. Real-time experiment for the mRNA expression of **e** anti- and **f** pro-inflammatory genes and **g** TREM-2; data are mean ± s.e.m. of four different experiments and expressed as fold-increased expression of 3xTg-AD mice vs. respective age-matched non-Tg mice (*p < 0.05 vs respective non-Tg value)

Skeletonized images obtained from confocal z-stack acquisitions (Fig. 3a, b) revealed that at 5–10 PNWs retinal 3xTg-AD microglia displayed increased arborization compared to non-Tg, while no differences were observed in the soma area (data not shown). Consistently, 3xTg-AD microglia cells are characterized by higher scanning domain (Fig. 3c, first panel), reflecting an increase of the arborization area with respect to the cell body area and suggesting differences in microglia basal scanning properties. Indeed, 3xTg-AD cells showed higher number of branches, branch points, and triple junctions compared to non-Tg cells, demonstrating that at pre-symptomatic stage 3xTg-AD microglia cells are more ramified (Fig. 3c, third and fourth panel). Longitudinal analysis during disease progression demonstrated that these differences disappeared in early-symptomatic mice, indicating similar morphology in 3xTg and non-Tg microglia (Fig. 3c, second panel). Conversely, in the retina of late-symptomatic AD mice, microglia became less ramified respect to non-Tg controls, with a morphological index becoming lower during disease progression (Fig. 3c).

It is known that the M/MΦ cell population in the brain is deeply involved in AD-related neurodegenerative process and its activation state changes during disease progression^28–30^; we thus analyzed microglia phenotype in the retina of pre-symptomatic and early-symptomatic AD mice and age-matched controls.

Microglia cells were stained using CD11b and CD45.2 antibodies and sorted by a FACSAriaIII; the number of microglia cells in the retina, identified as Cd11b + Cd45low cells (Fig. 3d), was comparable in the two genotypes (960 ± 150 cells in non-Tg, 1100 ± 100 in 3xTg-AD mice at 5–10 PNWs; 660 ± 100 non-Tg; 720 ± 150 3xTg-AD at 20–40 PNWs; *n* = 6 mice in each group, *p* = 0.1, two-way ANOVA) confirming immunofluorescence data (Fig. 2d). Data in Fig. 3e indicated that microglia cells sorted from the retina of 5–10 PNWs 3xTG-AD mice expressed higher level of some typical anti-inflammatory neuroprotective genes (Ym1 and CD206) respect to their age-matched controls. During disease progression, these genes undergo a significant reduction of expression in 3xTg-AD retina respect to their control. Conversely, pro-inflammatory, neurotoxic genes (iNOS, il1β), whose expression is low at pre-symptomatic AD stage, are remarkably increased during disease progression (Fig. 3f). It should be noted that this trend is not common to all the pro- and anti-inflammatory markers analyzed; in fact, TNF alpha (Fig. 3f) and Fizz-1 (Fig. 3e) showed opposite modulation during disease progression.

We also analyzed the expression level of the triggering receptor expressed on myeloid cells 2 (TREM-2), a marker expressed by microglia associated with neurodegenerative diseases^31^ in the retina. We found higher expression of TREM-2 mRNA in retinal microglia along the course of the disease (Fig. 3g), confirming, also in the 3xTg-AD model, the involvement of TREM-2 regulation in AD progression.

Altogether these data indicated that at pre-symptomatic AD stage, retinal microglia cells acquire a ramified, anti-inflammatory phenotype which can account for an increased tissue monitoring in response to first disease signs. During disease progression, retinal microglia showed a phenotype comparable to the pro-inflammatory one, with less ramified morphology, and neurodegenerative-associated markers, which can account for its neurotoxic effects.

### Extracellular and intracellular protein aggregates appear in the retina of pre-symptomatic mice

Alternative activation of retinal microglia toward an anti-inflammatory, neuroprotective phenotype in 3xTg-AD mice at pre-symptomatic stage, may reflect an increased tissue monitoring in response to early tissue damage, due to very early accumulation of AD-related protein aggregates.

**Fig. 4:**
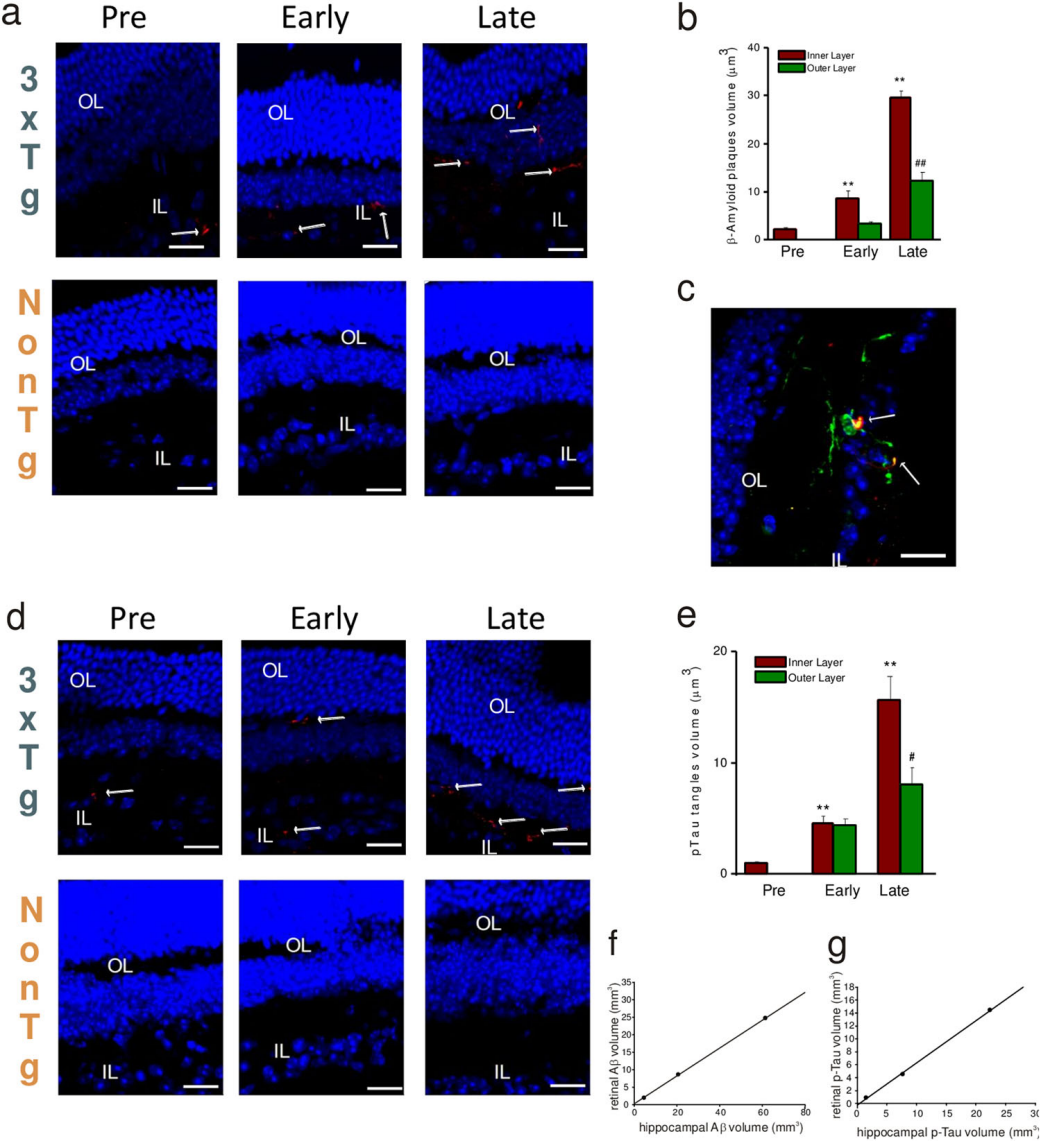
Amyloid beta plaques and pTau tangles are present in the retinas of pre-symptomatic 3xTg-AD mice. **a** Representative images of retinal slices immunolabeled with anti-Aβ antibody (red) and Hoechst for nuclei visualization (blue) at different ages of 3xTg-AD and non-Tg mice; plaque dimension was quantified as shown in **b** (**p < 0.01 vs pre in the inner layer; ^##^*p* < 0.01 vs early in the outer layer, *n* = 16 fields/four slices for each condition). **c** Representative image of retinal slice immunolabeled with anti-Aβ antibody (red), Iba1 (green), and Hoechst for nuclei visualization (blue), indicating that microglia is strictly connected to Aβ plaques emerging in retinal layers. **d** Representative images of retinal slice immunolabeled with anti-pTau antibody (red) and Hoechst for nuclei visualization (blue) at different ages of 3xTg-AD and non-Tg mice; tangles dimension was quantified as shown in **e** (**p < 0.01 vs pre in the inner layer; ^##^*p* < 0.01 vs early in the outer layer, *n* = 16 fields/four slices for each condition). **f** Correlation plot of Aβ plaque volume in hippocampal area (*x* axis) vs retina (*y* axis) of 3xTg-AD mice during disease progression; *n* = 16 fields/four slices for each condition, data are mean cumulative of different PNWs referred to the same AD stage; correlation coefficient: pre 0.531, early 1.00, late 0.916. **g** Correlation plot of pTau tangle volume in hippocampal area (*x* axis) vs retina (*y* axis) of 3xTg-AD mice during disease progression; *n* = 16 fields/four slices for each condition, data are mean cumulative of different PNWs referred to the same AD stage; correlation coefficient: pre 0.597, early 1.00, late 0.909

We analyzed Aβ accumulation in retinal samples from 3xTg-AD mice by means of immunofluorescence. As reported in Fig. 4a confocal analysis of Aβ staining revealed that plaques started to appear in the retina of 5–10 PNWs 3xTg-AD mice in the RGC layer (IL; minimum diameter: 580 nm), and that aggregate volume significantly increased during disease progression. Aβ staining was not observed in non-Tg retina of age-matched controls (Fig. 4a). Moreover, we found that in early-symptomatic mice, Aβ aggregated also in the outer layers (OL) of the retina, increasing their volume in late-stage mice (Fig. 4b). Co-immuno staining of Aβ and Iba1 revealed that in the retina of pre-symptomatic AD mice, microglia processes directly contacted Aβ plaques (Fig. 4c), suggesting that initial Aβ deposition is a signal-inducing enhanced microglia tissue monitoring.

Similarly, using a specific antibody against pTau, we detected pTau tangles in the RGC layer (IL) of 5–10 PNWs 3xTg-AD mice (minimum diameter: 680 nm). Tangle volume significantly increased (Fig. 4e) and tangles appeared in the outer layers (OL) of the retina during disease progression (Fig. 4d). No signal was found in non-Tg retinas (Fig. 4d). Moreover, as previously reported we found Aβ plaques and pTau tangles in hippocampal region already in pre-symptomatic mice, with volume increase during disease progression. Remarkably, the increase over time of Aβ plaques and pTau tangles volume was correlated with what was observed in the retina (Fig. 4f, g). Altogether, these data indicate that Aβ plaques and pTau tangles are detectable in the retina of young, pre-symptomatic mice, and contribute to glial activation and neurodegeneration.

## Discussion

Alzheimer’s disease represents nowadays the most important cause of dementia, with high social and economic costs; moreover, no specific cure counteracting neurodegeneration is currently available and some companies also retired their funding on AD-related clinical research. For these reasons, there is an urgent need to find new strategies for the diagnosis and the treatment of AD. In this framework, we consider retinal tissue as one of the best candidates that could recapitulate the AD-related neurodegenerative process happening in the brain, so we propose it as an easily attainable tissue for the early diagnosis and the follow-up of new therapies.

For this reason, we investigated the appearance of AD signs as neurodegeneration, intracellular and extracellular protein aggregates, and neuroinflammation, along the disease progression using a mouse model (3xTg-AD) which recapitulates most of the AD characteristics^21^.

We found, in 3xTg-AD mice, the presence of Aβ plaques and pTau tangles, ganglion neuron degeneration, astrogliosis and microglia activation, already at pre-symptomatic stage, making the retina a good candidate to be a window through which follow up the neurodegeneration process.

We here report, for the first time, that Aβ plaques and pTau tangles were present in the retina of young (5–20 PNWs) non-symptomatic 3xTg-AD mice in the RGC. Moreover, during disease progression (30–40 PMWs), in line with previous reports^13,32–34^, plaques and tangles appear also in the inner retinal layers.

Consistently with the appearance of plaques and tangles deposition, we show that neuronal apoptosis starts in the retina of young non-symptomatic 3xTg-AD mice, predominantly in RGC layer, and that the time course of neurodegeneration runs parallel to what is observed in the brain, thus making the presence of protein aggregates and neurodegeneration in the retina at non-symptomatic stage a possible pre-symptomatic AD biomarker.

We also analyzed retinal inflammatory microenvironment and the results herein indicate that glia (astrocytes and microglia) activation starts in the 3xTg-AD mouse retina at pre-symptomatic AD stage, with different activation timescales. Indeed, reactive GFAP-positive astrocytes are present in the 3xTg-AD retina already at 5–10 PNWs, much earlier than at 9 months, as previously reported^17,35^. Regarding microglia, we found that microglia density increased in 3xTg-AD retina only at late disease stages, while no changes were observed in non-Tg mice. On the other hand, microglia phenotype differs from control and AD mice already at 1–2 months of age. Indeed, we show that retinal microglia of young 3xTg-AD mice (5–10 PNWs) display, respect to their age-matched control, higher ramified morphology and expression of anti-inflammatory markers, suggesting microglia pre-activation with increased tissue monitoring and phagocytosis in response to first Aβ and pTau deposition in the RGC. We can speculate that different activation states of microglia cells may reflect in subtle morphological changes in branching and/or distribution and this could be due to the first appearance of protein aggregates that triggers microglia response to the disease. Making a longitudinal analysis during disease progression, we observed that in the early-symptomatic period (30–40 PNWs) retinal 3xTg-AD microglia acquired a less ramified morphology, in line with previously reported data^36^. This was paralleled, in our study, by a partial decrease of anti-inflammatory and increase of pro-inflammatory markers, together with TREM-2 upregulation, indicative of possible microglia switch from the homeostatic state to the neurodegenerative-associated one^31^.

Altogether, these data indicate that molecular and cellular determinants typical of AD are present in the retina of young 3xTg-AD mouse model, making the retina the ideal nervous tissue for early AD diagnosis and for studying AD pathology and treatment efficacy.

This observation may suggest the possibility that ocular biomarkers could be used for early detection of AD-associated neurodegeneration; however, whether the retina could be used to make a specific diagnosis of early, pre-symptomatic AD, and monitor AD progression, is still a matter of discussion. Indeed, while retinal examination performed as fundus images, following oral curcumin administration, revealed curcumin-positive retinal protein aggregate spots in live AD patients^37^, AD diagnosis still requires a combination of many biomarkers with a high degree of clinical signs to correlate cognitive deficits to retinal protein aggregates. Moreover, it should be considered that (i) curcumin binding is not specific for Aβ, (ii) amyloid aggregates are found also in macular degeneration^38^, and (iii) available retinal fundus examination does not allow the detection of structures in the sub-micrometric scale. Indeed, as shown for 3xTg-AD mice, the use of protein aggregates (Aβ and pTau) as early diagnostic biomarkers requires a submicron lateral resolution (see results and Fig. 4). Thus, a huge improvement is needed in developing both specific Aβ and pTau ligands and long-working distance high-resolution imaging techniques to make possible a non‐invasive and inexpensive diagnosis of AD through the retinal scan in live patients.

## Electronic supplementary material

Supplementary Figure 1

Supplementary Table 1
