# Supplementary figures and images for: Inflammation, neurodegeneration and protein aggregation in the retina as ocular biomarkers for Alzheimer’s disease in the 3xTg-AD mouse model
Source: Cell Death Dis. 2018 Jun 7;9(6):685. doi: 10.1038/s41419-018-0740-5 (PMC5992214; doi:10.1038/s41419-018-0740-5)

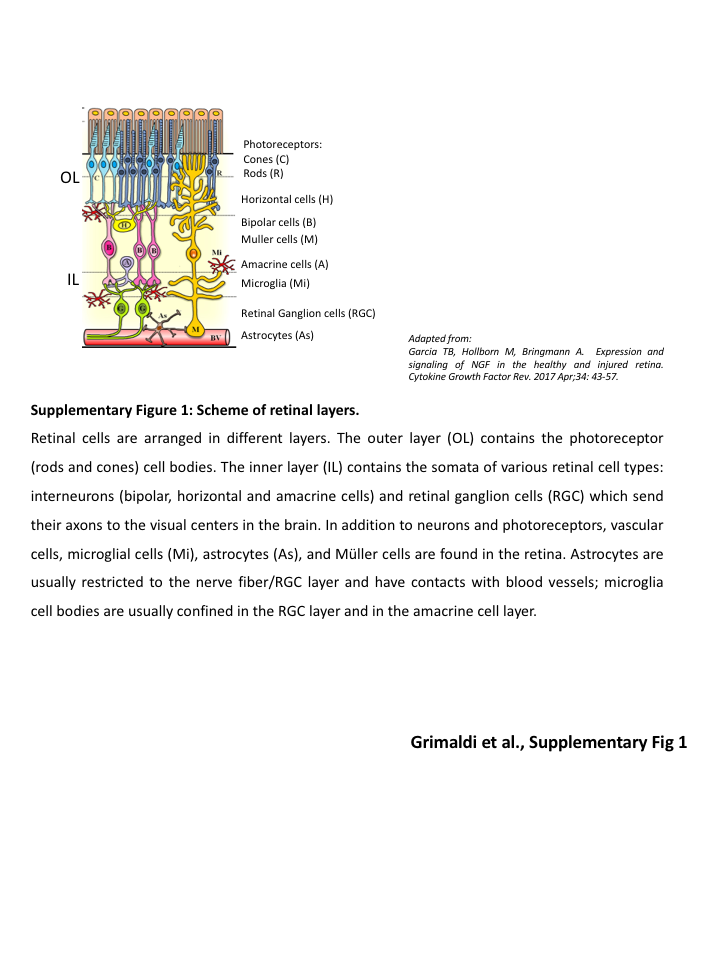

Supplement: Supplementary file 1 — Supplementary Figure 1 [file 41419_2018_740_MOESM1_ESM.tif]
