# Supplementary material for: Inflammation, neurodegeneration and protein aggregation in the retina as ocular biomarkers for Alzheimer’s disease in the 3xTg-AD mouse model
Source: Cell Death Dis. 2018 Jun 7;9(6):685. doi: 10.1038/s41419-018-0740-5 (PMC5992214; doi:10.1038/s41419-018-0740-5)
Supplement: Supplementary file 2 — Supplementary Table 1 [file 41419_2018_740_MOESM2_ESM.doc]

**Supplementary Table 1. Primers used for Real Time-PCR analysis.**

| **Gene** | **Primer Forward (5’-3’)** | **Primer Reverse (5’-3’)** |
| --- | --- | --- |
| *cd206*  *fizz1*  *gapdh*  *il1b*  *inos*  *tnfa*  *ym1*  *trem-2* | CAAGGAAGGTTGGCATTTGT  CCAATCCAGCTAACTATCCCTCC  TCGTCCCGTAGACAAAATGG  GCAACTGTTCCTGAACTCAACT  ACATCGACCCGTCCACAGTAT  GTGGAACTGGCAGAAGAG  CAGGTCTGGCAATTCTTCTGAA  ACAGCACCTCCAGGAATCAAG | CCTTTCAGTCCTTTGCAAGT  ACCCAGTAGCAGTCATCCCA  TTGAGGTCAATGAAGGGGTC  ATCTTTTGGGGTCCGTCAACT  CAGAGGGGTAGGCTTGTCTC  CCATAGAACTGATGAGAGG  GTCTTGCTCATGTGTGTAAGTGA  CCACAGCCCAGAGGATGC |

Grimaldi et al., Suppl Tab 1
